# Comparison of dose–volume histograms between proton beam and X-ray conformal radiotherapy for locally advanced non-small-cell lung cancer

**DOI:** 10.1093/jrr/rru082

**Published:** 2014-11-03

**Authors:** Toshiki Ohno, Yoshiko Oshiro, Masashi Mizumoto, Haruko Numajiri, Hitoshi Ishikawa, Toshiyuki Okumura, Toshiyuki Terunuma, Takeji Sakae, Hideyuki Sakurai

**Affiliations:** Departments of Radiation Oncology and Proton Medical Research Center, University of Tsukuba, 1-1-1 Tennoudai, Tsukuba, Ibaraki, 305-8575, Japan

**Keywords:** proton therapy, locally advanced NSCLC, dose escalation, DVH

## Abstract

The purpose of this study was to compare the parameters of the dose–volume histogram (DVH) between proton beam therapy (PBT) and X-ray conformal radiotherapy (XCRT) for locally advanced non-small-cell lung cancer (NSCLC), according to the tumor conditions. A total of 35 patients having NSCLC treated with PBT were enrolled in this analysis. The numbers of TNM stage and lymph node status were IIB (*n* = 3), IIIA (*n* = 15) and IIIB (*n* = 17), and N0 (*n* = 2), N1 (*n* = 4), N2 (*n* = 17) and N3 (*n* = 12), respectively. Plans for XCRT were simulated based on the same CT, and the same clinical target volume (CTV) was used based on the actual PBT plan. The treatment dose was 74 Gy-equivalent dose (GyE) for the primary site and 66 GyE for positive lymph nodes. The parameters were then calculated according to the normal lung dose, and the irradiation volumes of the doses (Vx) were compared. We also evaluated the feasibility of both plans according to criteria: V5 ≥ 42%, V20 ≥ 25%, mean lung dose ≥ 20 Gy. The mean normal lung dose and V5 to V50 were significantly lower in PBT than in XCRT. The differences were greater with the more advanced nodal status and with the larger CTV. Furthermore, 45.7% of the X-ray plans were classified as inadequate according to the criteria, whereas 17.1% of the proton plans were considered unsuitable. The number of inadequate X-ray plans increased in cases with advanced nodal stage. This study indicated that some patients who cannot receive photon radiotherapy may be able to be treated using PBT.

## INTRODUCTION

Radiotherapy plays an important role in the treatment of locally advanced, unresectable non-small-cell lung cancer (NSCLC). In order to achieve the maximum survival benefit with radiotherapy, the dose–response relationship and its combination with chemotherapy has been investigated since the 1980s. Several successful dose-escalation studies with concurrent chemotherapy have been undertaken worldwide and have led to improved tumor control and survival at doses above 70 Gy [1–8]. However, the Phase III study by RTOG showed no survival benefit with a dose of 74 Gy compared with 60 Gy [9]. While Cox *et al.* reported that pulmonary or cardiopulmonary effects of radiotherapy could affect the outcome, the reason for this was unclear [10]. Meanwhile, Chang *et al.* successfully administered chemo-proton therapy for unresectable Stage III NSCLC with a dose of 74 GyE, and reported a median survival time of 29.4 months [11]. We consider that PBT will be key to safe dose escalation for locally advanced NSCLC due to the sharp energy peak, called the Bragg peak.

The dosimetric comparison of protons and photon radiotherapy for early stage NSCLC has been widely discussed, and some reports of early-stage NSCLC have shown that PBT also significantly reduces the normal lung dose [12–17]. However, there have been few investigations of the differences in dose distribution for advanced NSCLC [17, 18].

In this report, we simulated proton therapy using a high radiation dose at 74 GyE for unresectable locally advanced NSCLC and compared the parameters of the dose–volume histograms (DVHs) for PBT and photon conformal radiotherapy (XCRT), based upon the tumor condition, i.e. stage, lymph node status, and target volume.

## MATERIALS AND METHODS

### Patient characteristics

A total of 35 cases of inoperable locally advanced Stage IIB and III NSCLC were enrolled in this analysis. The TNM stage was Stage IIB in three patients, IIIA in 15, and IIIB in 17. The nodal stage was N0, N1, N2 and N3 in 2, 4, 17 and 12 patients, according to the TNM classification of malignant tumors, sixth edition. The tumor was located in the upper lobe in 24 patients and in the middle and lower lobe in 11 patients. All patients were treated with proton beams of 155–250 MeV generated using a synchrotron accelerator (Hitachi Inc., Ibaraki, Japan) at the Proton Medical Research Center. This study was approved by our institutional review board, and written informed consent was obtained from all patients.

### Treatment planning

For treatment planning, chest CT images were obtained in 5-mm thick slices, with the patient in a body cast in the treatment position (Engineering System Co., Matsumoto, Japan), during the end-expiratory phase using a respiratory-gated system (DAR-3000, Shimadzu, Kyoto, Japan). The dose calculation for PBT and XCRT was performed using the same CT series for each patient with the pencil beam method for PBT (proton treatment planning software ver. 2, Hitachi Inc., Ibaraki, Japan) and with superposition on for XCRT (Xio ver. 4, Elekta, Stockholm, Sweden). Proton beams of 155–250 MeV and X-ray irradiation of 10 MV were used in the treatment plans. The treatment planning system for PBT automatically estimated the conditions required for beam delivery, which include a ridge filter, a range shifter, a collimator and bolus. The beam delivery system created a homogeneous dose distribution at the prescription dose using the spread-out Bragg peak of the proton beams. The concept of dose delivery, for both the target and normal tissues, was exactly the same for PBT as for XCRT; the daily fractionation dose was 2 Gy, and the primary site and positive lymph nodes were irradiated at 74 Gy and 66 Gy, respectively.

We defined the clinical target volume (CTV) as the primary tumor and clinically positive lymph nodes. Prophylactic lymph nodes were not included in the CTV. Clinically positive lymph nodes were defined as nodes ≥1 cm as visualized on a CT scan or as PET-positive lymph nodes. CTV-p was defined as the primary tumor alone. The planned target volume (PTV) and PTV-p encompassed the CTV and CTV-p, respectively, with a 5–10-mm margin in all directions and an additional 5-mm margin in the caudal direction (to compensate for respiratory motion), and the coverage of PTVs was provided for by more than 95% prescribed doses. To ensure this coverage, we set up ∼5-mm distal and proximal margins for PTVs at PBT. The total normal lung volume was the total lung volume reduced by the tumor volume (gross tumor volume: GTV) and atelectasis. The median CTV was 228.5 cm^3^ (range: 34.4–555.5 cm^3^), and the median total normal lung volume was 3426.4 cm^3^ (range: 1219–5179 cm^3^).

**Fig. 1.**
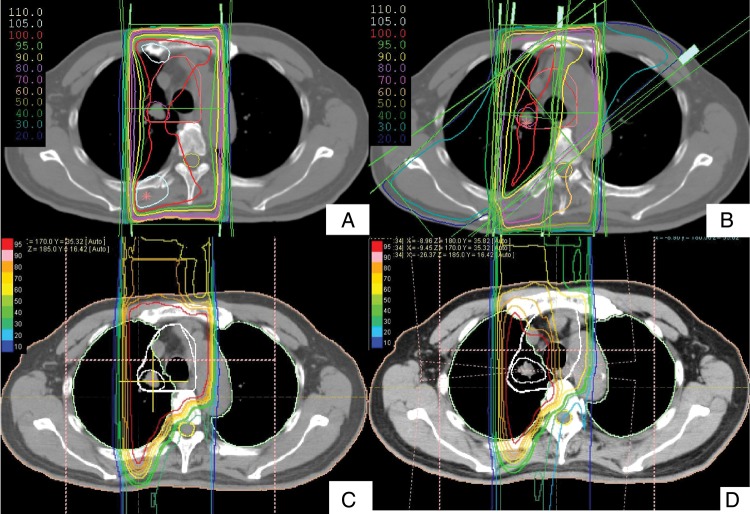
Comparison of dose distributions for T1N3M0 lung cancer between XCRT (A/B) and PBT (C/D). (**A**) An initial 44 Gy of XCRT was delivered via the anterior and posterior ports. Note the difference in dose to the spinal cord between XCRT and PBT. (**B**) Sum plan of XCRT. After 44 Gy, an oblique field was needed to avoid the spinal cord in XCRT. (**C**) In PBT, a reduction of the dose to the spinal cord to less than 50% allows using the anterior and posterior ports until 66 GyE to the CTV1. (**D**) Sum plan of PBT.

For PBT, both 66 GyE and an additional 8 GyE were delivered via two to three ports in the optimal direction to maintain a tolerable spinal dose (∼40 GyE) to PTV and PTV-p, respectively. For XCRT, an initial 44 Gy dose was delivered via the anterior and posterior ports for PTV, and 22 Gy was then irradiated using oblique fields to avoid the spinal cord. Finally, we applied a booster dose at 8 Gy to PTV-p. A typical treatment plan of XCRT and PBT is shown in Fig. 1.

### Analysis

The DVH of the lung was calculated during planning for both PBT and XCRT, and the relationship between tumor factor (TNM stage, T stage, N stage, CTV) and dosimetric factors (i.e. mean lung dose (MLD) and the percentage volume of the whole lung receiving more than a certain dose (Vx)) were analyzed by a two-sample *t*-test and the correlation coefficient. We also evaluated the feasibility of the plans according to the criteria reported for the increasing risk of radiation pneumonitis, as follows: V5 ≥ 42% [19], V20 ≥ 25% [20], MLD ≥ 20 Gy [21].

All statistical analyses were performed using statistical software (SPSS, IBM Inc., NY, USA), and *P*-values < 0.05 were considered statistically significant.

## RESULTS

### Mean lung dose

**Fig. 2.**
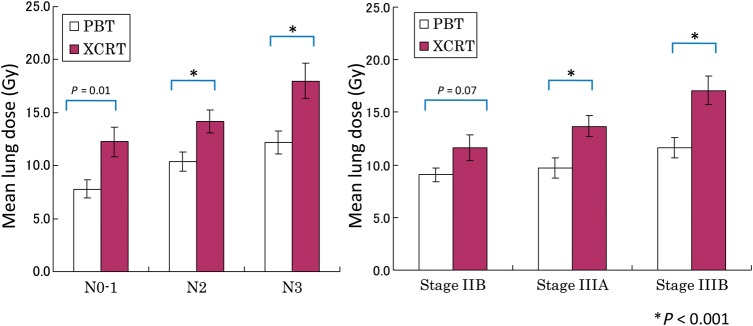
The relationship between the mean lung dose and N stage for each modality of PBT and XCRT.

**Fig. 3.**
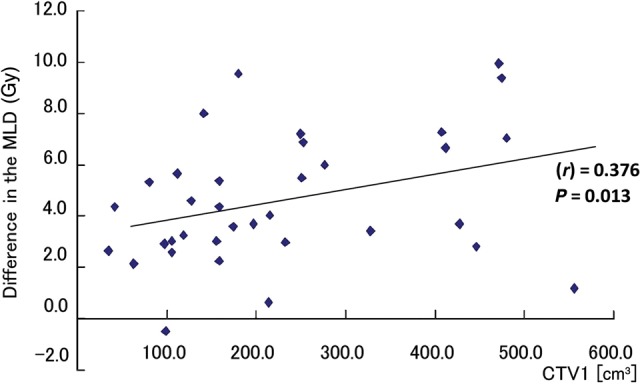
The correlation between CTV1 and the reduction in MLD. Difference in MLD = MLD (XCRT) − MLD (PBT).

The relationship between the MLD and lymph node status or stage is shown in Fig. 2. The average MLD for N0–1, N2, N3 in PBT and XCRT was 7.80 Gy vs 12.25 Gy (*P* = 0.01), 10.41 Gy vs 14.17 Gy (*P* < 0.001), and 12.20 Gy vs 18.00 Gy (*P* < 0.001), and the average MLD for Stage IIB, IIIA and IIIB was 9.05 Gy vs 11.61 Gy (*P* = 0.07), 9.70 Gy vs 13.68 Gy (*P* < 0.001) and 11.62 Gy vs 17.08 Gy (*P* < 0.001), respectively. The MLD in the PBT was significantly lower than that of XCRT for all stages and nodal status. The CTV volume was also a significant factor affecting MLD (coefficient factor (*r*) = 0.376, *P* = 0.013) (Fig. 3). The larger the CTV, the greater the difference in MLD between the PBT and the XCRT plans.

### Lung volume receiving more than a certain dose (Vx)

**Fig. 4.**
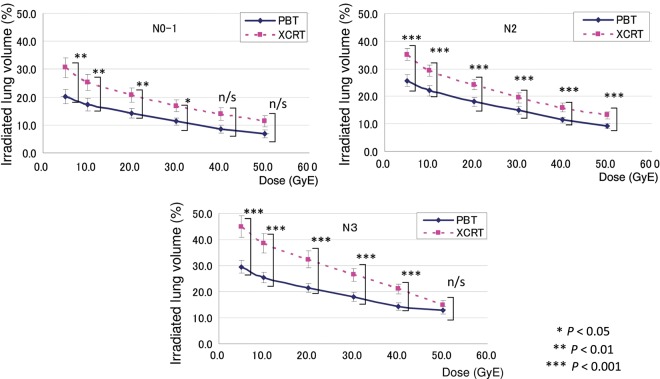
The relationship between V5–50 and N stage for each modality of PBT and XCRT.

**Fig. 5.**
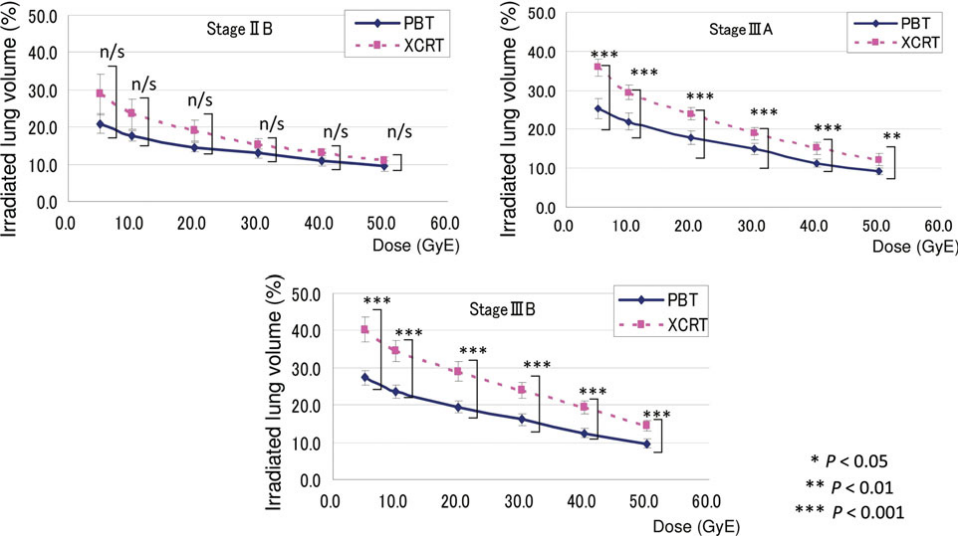
The relationship between V5–50 and TNM stage for each modality of PBT and XCRT.

The results of V5, V10, V20, V30, V40 and V50 in accordance with nodal stages are shown in Fig. 4. The irradiated normal lung volume increased significantly with the advanced nodal stage. Furthermore, in Fig. 4, both lines in the PBT and XCRT appear to be nearly parallel in the N0 to N2 patients, but not in the N3 patients. This means that the differences in the lung doses between the XCRT and PBT are greater in the N3 patients compared with the N0–2 patients, especially for the dose to the lower to middle lung lobes. The irradiated normal lung volume also increased significantly with the advanced TNM stage (Fig. 5). The correlation between CTV and the differences in Vx (Vx in XCRT – Vx in PBT) was also observed in V30–V50 (*P* = 0.391, 0.454, 0.266, 0.046, 0.019 and 0.030 for V5, V10, V20, V30, V40 and V50, respectively). Thus, the differences between PBT and XCRT were observed, and while the differences were greater at lower doses, the correlation of Vx differences with CTV was stronger for larger doses; i.e. V30–V50.

### Feasibility of the plan

Table 1 summarizes the number of inadequate plans for photon radiotherapy and PBT. According to the criteria of V5 ≥ 42% [19], V20 ≥ 25% [20] and MLD ≥ 20 Gy([21], 45.7% of the XCRT plans were classified as inadequate, whereas only 17.1% of the proton plans were not suitable. The number of inadequate XCRT plans increased accordingly with the advanced nodal stage.

**Table 1.** The numbers of inadequate plan in XCRT and PBT according to the criteria of V5 ≥ 42% [19], V20 ≥ 25% [20] and MLD ≥ 20 Gy [21]

Group	XCRT	PBT	*P-value*
All (*n* = 35)	16 (45.7%)	6 (17.1%)	0.01
N0–1 (*n* = 6)	1 (16.7%)	0 (0.0%)	
N2 (*n* = 17)	5 (29.4%)	2 (11.8%)	
N3 (*n* = 12)	10 (83.3%)	4 (33.3%)	0.013

## DISCUSSION

Radiation pneumonitis is a significant concern during radiotherapy for patients with lung cancer. The risk of radiation pneumonitis correlates closely with the volume dose of the normal lung. Tsujino *et al.* found that V20 correlated significantly with the incidence of radiation pneumonitis. They reported that the incidence of severe radiation pneumonitis was significantly higher in patients with V20 ≥ 25% [20]. Marks *et al.* analyzed the findings of previous studies and suggested that an MLD of 20–23 Gy with conventional fractions was appropriate to limit the risk of radiation pneumonitis to ≤ 20% [21]. Furthermore, Wang *et al.* analyzed patients with NSCLC that were treated with concurrent chemoradiotherapy and showed a significantly lower frequency of Grade 3 or worse radiation pneumonitis for patients with V5 ≤ 42% compared with those patients with V5 > 42% [19]. Therefore, radiotherapy can be more difficult for larger tumors, with increasing risk of radiation pneumonitis in the treatment of locally advanced NSCLC.

Chemoradiotherapy is now standard treatment for unresectable locally advanced NSCLC. However, the feasible doses for concurrent chemoradiotherapy remain controversial. Even though a Phase III study (RTOG 0617) was not able to show any survival benefit by dose escalation, the toxicities were considered tolerable, and survival was improved in many prospective studies [1–3, 8].

Meanwhile, proton beams are now popular for various cancers because of their excellent dose localization, and they can be applied to many patients with a variety of malignancies. Some authors have reported favorable results for PBT for advanced NSCLC [22–24]. Chang *et al.* reported that the median survival for patients with Stage III NSCLC was 29.4 months with concurrent chemo–proton therapy using a dose of 74 GyE, with no Grade 4 non-hematologic toxicities [22]. Oshiro *et al.* reported that while the median survival was 21.3 months, Grade ≥ 3 lung toxicities were observed in three patients, and no severe esophagitis was observed in the standalone PBT for 57 patients with Stage III NSCLC [24]. These results suggest that PBT has a great potential for producing a survival benefit with less toxicities, which may be a result of its excellent dose localization, as noted above. To the best of our knowledge, there have only been two reports suggesting dosimetric advantages for PBT in advanced NSCLC. Chang *et al*. compared dose distribution in XCRT (63 Gy) with PBT (74 GyE) plans and reported that V5, V10 and V20 were significant lower in PBT plans. Stuschke *et al.* compared intensity-modulated proton therapy (IMPT), photon intensity-modulated radiotherapy (IMXT) and tomotherapy in six patients and found that MLD and V10 and V20 were lowest for the IMPT plans [18]. Our study also showed dosimetric advantages of proton compared with photon radiotherapy in the treatment of advanced NSCLC, especially for more advanced lymph node stages, and some patients who received PBT could not be treated with photon radiotherapy. Furthermore, a significant correlation was revealed between the CTV and MLD, V30, V40 and V50 in our study, which suggested that PBT is more advantageous for a larger CTV to reduce doses to the normal lung, especially for critical doses >20 Gy. Thus, PBT appears to be more advantageous for patients with more advanced NSCLC, and can provide treatment opportunities for some patients with fewer options.

However, there are some limitations to our study. While DVHs were investigated in the initial plan, some plans were changed practically as a consequence of tumor shrinking. Furthermore, the calculation algorithm differed between PBT and photon radiotherapy in this study. Our results reflect a practical propensity for a dose–volume relationship, but comparison of adoptive plans after refinement (using a Monte Carlo algorithm) will be necessary for precise analysis in the future.

In conclusion, PBT can reduce the normal lung dose compared with XCRT, especially in the advanced nodal stage, and more locally advanced patients can be treated by this modality using PBT.

## FUNDING

This research was partly supported by the Funding Program for World-Leading Innovative R&D on Science and Technology (FIRST Program) initiated by the Council for Science and Technology Policy (CSTP). This work was also supported in part by a Grant-in-Aid from the Ministry of Education, Science, Sports and Culture of Japan. Funding to pay the Open Access publication charges for this article was provided by Proton Medical Research Center, University of Tsukuba.
